# Extra Professional Duties

**Published:** 1833

**Authors:** 


### II. EXTRA PROFESSIONAL DUTIES.

Among the obligations imposed on medical men in this new country, by their belonging to a *learned* profession, is that of encouraging education generally. This is a duty of every man of letters in society, and physicians are or ought to be of this *caste*. They should know and feel, moreover, that for the profession to attain to the dignity of sound scholarship, students of medicine should be well taught and disciplined in their preparatory education; and hence it becomes, in part, a professional duty for physicians to promote, by every means in their power, the academical and collegiate education of our sons, from among whom the ranks of the profession are to be replenished. It is under this conviction, that we write this article, not merely to discharge our own duty, but to stimulate our brethren in the West to the discharge of theirs. But let us to the point.

For the advancement of the interests of education in the valley of the Mississippi, a number of teachers, two or three years since, formed an association under the name of the "Western Literary Institute and College of Professional Teachers;" and on the 9th ultimo, they held in Cincinnati, their second annual meeting. It was attended by gentlemen from Kentucky, Illinois and Ohio; among whom were a number of professors, and the presidents of four colleges or universities. A week was spent in consultation and the adoption of various resolutions, designed to promote common schools, academical and collegiate education. There were several public lectures delivered daily, on a variety of important topics, which were listened to with an auspicious attention by large and intelligent audiences. In the course of the week, measures which promise well, were devised and adopted, for effecting in this city, on the second Monday in April next, a numerous convention of literary and scientific men, from all the Mississippi states, to confer on the means of giving a simultaneous impulse to the cause of education in the great valley, which we hope, for the honor of the profession, will be approved and attended by such of its physicians, as may at the time be able to absent themselves from their business. The committee appointed to bring about this meeting, and prepare matters for its consideration, are the Rev. Lyman Beecher, President of the Lane Seminary, near Cincinnati; Mr. Thomas J. Matthews, President of the Woodward High School, in this city; the Rev. B. O. Peers, President of Transylvania University; Nathan Guilford, John P. Foote, J. D. Garrard, and James Hall, Esquires, of this place, gentlemen in all respects qualified for the undertakiug confided to them.

Meanwhile, a convention of teachers and professors for the state of Kentucky, is to be held at Lexington, in the first week of November, when the public Commencement of the collegiate department of Transylvania will be held, and the introductory lectures of the medical and law professors be delivered. It is expected that many of the past graduates of all the departments will then revisit their *alma mater.*

We congratulate society, and particularly our own profession, on these movements; believing as we do, that they are the commencement of that systematic and concentrated effort, which is essential to the proper elevation of the intellectual character of this great region; so rich in national resources and physical power, but so poor in the means of literar y and scientific culti vation—we mean good teachers of all grades. When the opportunity of a better academical education especially, shall be within the reach of our youth generally, our students of medicine will feel ashamed to hand in theses remarkable for nothing but violations of spelling and grammar; and our physicians not be kept from recording and publishing their observations, because they do not know how to express themselves intelligibly.

